# Ethyl 2-{5-[(3-oxo-3,4-di­hydro-2*H*-1,4-benzo­thia­zin-4-yl)meth­yl]-1*H*-1,2,3-triazol-1-yl}acetate

**DOI:** 10.1107/S1600536813034697

**Published:** 2014-01-08

**Authors:** Nada Kheira Sebbar, Abdelfettah Zerzouf, El Mokhtar Essassi, Mohamed Saadi, Lahcen El Ammari

**Affiliations:** a Laboratoire de Chimie Organique Hétérocyclique URAC 21, Pharmacochimie, Av Ibn Battouta, BP 1014, Faculté des Sciences, Université Mohammed V-Agdal, Rabat, Morocco; b Laboratoire de Chimie Organique et Etudes Physico-chimique, ENS Takaddoum, Rabat, Morocco; c Laboratoire de Chimie du Solide Appliquée, Faculté des Sciences, Université Mohammed V-Agdal, Avenue Ibn Battouta, BP 1014, Rabat, Morocco

## Abstract

In the title compound, C_15_H_16_N_4_O_3_S, the six-membered heterocycle of the benzo­thia­zine fragment exhibits a screw boat conformation. The dihedral angle between the planes through the triazole ring and the benzene ring fused to the 1,4-thia­zine ring is 62.98 (11)°. The mean plane formed by the atoms belonging to the acetate group is nearly perpendicular to the triazole ring [dihedral angle = 74.65 (12)°]. In the crystal, mol­ecules are linked by pairs of C—H⋯O inter­actions, forming dimeric aggregates.

## Related literature   

For the pharmacological activity of benzo­thia­zine derivatives, see: Fringuelli *et al.* (1998 ▶); Lopatina *et al.* (1982 ▶); Rathore & Kumar (2006 ▶). For related structures, see: Keita *et al.* (2000 ▶); Zerzouf *et al.* (2001 ▶); Barryala *et al.* (2011 ▶). For puckering calculation see: Cremer & Pople (1975 ▶).
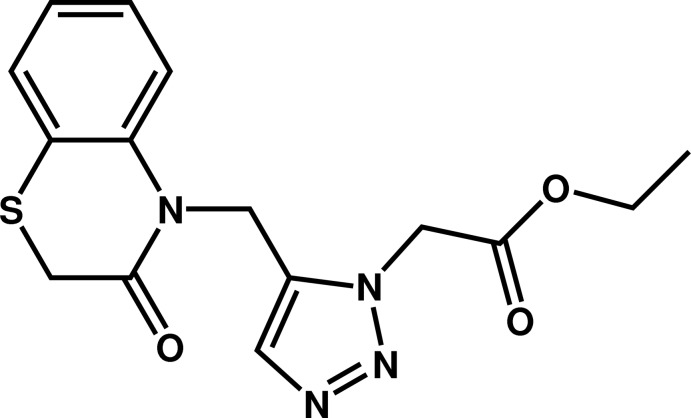


## Experimental   

### 

#### Crystal data  

C_15_H_16_N_4_O_3_S
*M* *_r_* = 332.38
Triclinic, 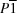

*a* = 5.6414 (2) Å
*b* = 11.1604 (4) Å
*c* = 13.3724 (5) Å
α = 73.823 (2)°
β = 87.226 (2)°
γ = 88.566 (2)°
*V* = 807.59 (5) Å^3^
*Z* = 2
Mo *K*α radiation
μ = 0.22 mm^−1^
*T* = 296 K
0.37 × 0.34 × 0.28 mm

#### Data collection  

Bruker X8 APEX diffractometer
Absorption correction: multi-scan (*SADABS*; Bruker, 2009 ▶) *T*_min_ = 0.692, *T*_max_ = 0.747
16305 measured reflections
3560 independent reflections
2963 reflections with *I* > 2σ(*I*)
*R*_int_ = 0.028

#### Refinement  

*R*[*F* ^2^ > 2σ(*F* ^2^)] = 0.045
*wR*(*F* ^2^) = 0.124
*S* = 1.05
3560 reflections
208 parameters
H-atom parameters constrained
Δρ_max_ = 0.39 e Å^−3^
Δρ_min_ = −0.32 e Å^−3^

### 

Data collection: *APEX2* (Bruker, 2009 ▶); cell refinement: *SAINT* (Bruker, 2009 ▶); data reduction: *SAINT*; program(s) used to solve structure: *SHELXS97* (Sheldrick, 2008 ▶); program(s) used to refine structure: *SHELXL97* (Sheldrick, 2008 ▶); molecular graphics: *ORTEP-3 for Windows* (Farrugia, 2012 ▶); software used to prepare material for publication: *PLATON* (Spek, 2009 ▶) and *publCIF* (Westrip, 2010 ▶).

## Supplementary Material

Crystal structure: contains datablock(s) I. DOI: 10.1107/S1600536813034697/tk5283sup1.cif

Structure factors: contains datablock(s) I. DOI: 10.1107/S1600536813034697/tk5283Isup2.hkl

Supporting information file. DOI: 10.1107/S1600536813034697/tk5283Isup3.cml

CCDC reference:

Additional supporting information:  crystallographic information; 3D view; checkCIF report

## Figures and Tables

**Table 1:** Hydrogen-bond geometry (Å, °)

*D*—H⋯*A*	*D*—H	H⋯*A*	*D*⋯*A*	*D*—H⋯*A*
C4—H4⋯O2^i^	0.93	2.59	3.445 (3)	154

Symmetry code: (i) 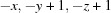.

**Fig. 1.**
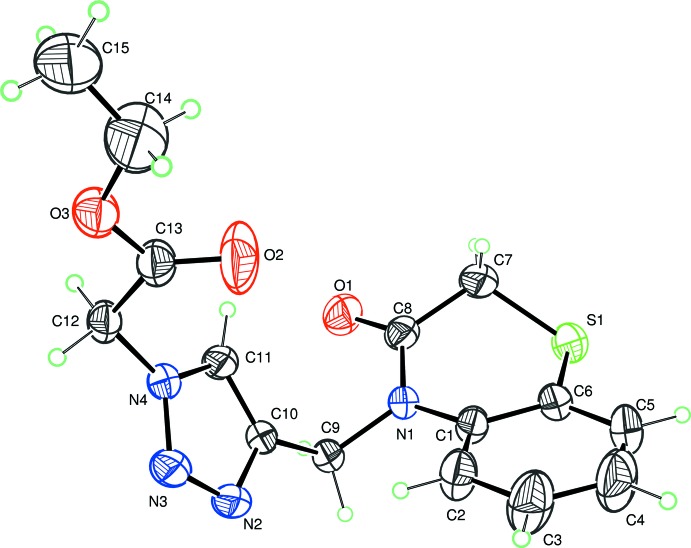
Molecular structure of the title compound with the atom-labelling scheme. Displacement ellipsoids are drawn at the 50% probability level. H atoms are represented as small circles.

**Fig. 2.**
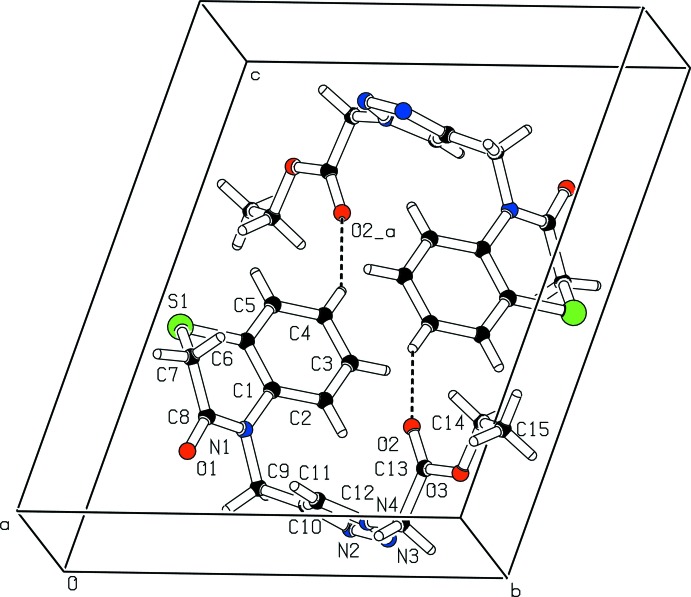
Three dimensional plot of the title compound, showing molecules linked through C4–H4···O2 hydrogen bonds (dashed lines).

## supplementary crystallographic information

### 2.1. Synthesis and crystallization

To a solution of 4-(prop-2-yn-1-yl)-2*H*-1, 4-benzo­thia­zin-3-one (0.2 g, 0.7 mmol) in ethanol (15 ml) was added azide ethyl acetate (0.20 ml, 1.89 mmol). The mixture was stirred under reflux for 24 h. After completion of reaction (monitored by TLC), the solution was concentrated and the residue was purified by column chromatography on silica gel by using a mixture (hexane/ethyl acetate 2/1). Crystals were obtained when the solvent was allowed to evaporate. The solid product was purified by recrystallization from ethanol to afford yellow crystals in 75% yield.

### 2.2. Refinement

The H atoms were located in a difference map and treated as riding with C—H = 0.93 Å (aromatic), C—H = 0.97 Å (methyl­ene) and C—H = 0.96 Å, (methyl), and with *U*_iso_(H) = 1.2 *U*_eq_ (methyl­ene and ammonium) and *U*_iso_(H) = 1.5 *U*_eq_ for the methyl. Owing to poor aggreement, three reflections, *i.e*. (0 1 1), (0 1 0) and (0 0 1), were omitted from the final cycles of refinement.

### 3. Results and discussion

Several derivatives of benzo­thia­zines form an important class of bioactive molecules in the field of drugs and pharmaceuticals. Applications in various therapeutic areas, include their use as anti-depressants (Lopatina *et al.*, 1982); anti-fungals (Fringuelli *et al.*, 1998) and anti-microbials (Rathore & Kumar, 2006). The present work is a continuation of the investigation of the benzo­thia­zine derivatives published recently by our team (Keita *et al.*, 2000; Zerzouf *et al.*, 2001; Barryala *et al.*, 2011). The aim of the present paper was to study the recently synthesized ethyl2-{5-[(3-oxo-2*H*-1,4-benzo­thia­zin-4-yl)methyl]- 1,2,3-triazol-1-yl}acetate crystal structure by X-ray crystallography at room temperature.

The molecule of the title compound is build up from two fused six-membered rings linked to a triazole ring which is attached to an ethyl­acetate group as shown in Fig.1. The 1,4-thia­zine ring adopts a screw boat conformation as indicated by the puckering amplitude Q = 0.6536 (17) Å, and spherical polar angle θ = 112.04 (16)°, with φ = 152.14 (18)° (Cremer & Pople, 1975). The benzene ring (C1 to C6) makes a dihedral angle of 62.98 (11)° with the triazole ring (N2N3N4C10C11) which is nearly perpendicular to the mean plane through the acetate atoms (C12C13O2O3) as indicated by the dihedral angle between them of 74.65 (12)°. In the crystal, the molecules are linked by weak inter­molecular C4–H4···O2 inter­actions to form dimers (see Fig.2 and Table 1).

### Crystal data

**Table:**

C_15_H_16_N_4_O_3_S	*Z* = 2
*M**_r_* = 332.38	*F*(000) = 348
Triclinic, *P*1	*D*_x_ = 1.367 Mg m^−^^3^
*a* = 5.6414 (2) Å	Mo *K*α radiation, λ = 0.71073 Å
*b* = 11.1604 (4) Å	Cell parameters from 3560 reflections
*c* = 13.3724 (5) Å	θ = 2.8–27.1°
α = 73.823 (2)°	µ = 0.22 mm^−^^1^
β = 87.226 (2)°	*T* = 296 K
γ = 88.566 (2)°	Block, yellow
*V* = 807.59 (5) Å^3^	0.37 × 0.34 × 0.28 mm

### Data collection

**Table:**

Bruker X8 APEX diffractometer	3560 independent reflections
Radiation source: fine-focus sealed tube	2963 reflections with *I* > 2σ(*I*)
Graphite monochromator	*R*_int_ = 0.028
φ and ω scans	θ_max_ = 27.1°, θ_min_ = 2.8°
Absorption correction: multi-scan (*SADABS*; Bruker, 2009)	*h* = −7→7
*T*_min_ = 0.692, *T*_max_ = 0.747	*k* = −14→14
16305 measured reflections	*l* = −17→16

### Refinement

**Table:**

Refinement on *F*^2^	Primary atom site location: structure-invariant direct methods
Least-squares matrix: full	Secondary atom site location: difference Fourier map
*R*[*F*^2^ > 2σ(*F*^2^)] = 0.045	Hydrogen site location: inferred from neighbouring sites
*wR*(*F*^2^) = 0.124	H-atom parameters constrained
*S* = 1.05	*w* = 1/[σ^2^(*F*_o_^2^) + (0.0523*P*)^2^ + 0.3602*P*] where *P* = (*F*_o_^2^ + 2*F*_c_^2^)/3
3560 reflections	(Δ/σ)_max_ < 0.001
208 parameters	Δρ_max_ = 0.39 e Å^−^^3^
0 restraints	Δρ_min_ = −0.32 e Å^−^^3^

### Special details

**Table:**

Geometry. All s.u.'s (except the s.u. in the dihedral angle between two l.s. planes) are estimated using the full covariance matrix. The cell s.u.'s are taken into account individually in the estimation of s.u.'s in distances, angles and torsion angles; correlations between s.u.'s in cell parameters are only used when they are defined by crystal symmetry. An approximate (isotropic) treatment of cell s.u.'s is used for estimating s.u.'s involving l.s. planes.
Refinement. Refinement of *F*^2^ against all reflections. The weighted *R*-factor *wR* and goodness of fit *S* are based on *F*^2^, conventional *R*-factors *R* are based on *F*, with *F* set to zero for negative *F*^2^. The threshold expression of *F*^2^ > 2σ(*F*^2^) is used only for calculating *R*-factors(gt) *etc*. and is not relevant to the choice of reflections for refinement. *R*-factors based on *F*^2^ are statistically about twice as large as those based on *F*, and *R*- factors based on all data will be even larger.

### Fractional atomic coordinates and isotropic or equivalent isotropic displacement parameters (Å^2^)

**Table:**

	*x*	*y*	*z*	*U*_iso_*/*U*_eq_	
C1	0.0414 (3)	0.32734 (16)	0.35494 (13)	0.0404 (4)	
C2	−0.1005 (4)	0.43140 (19)	0.35277 (16)	0.0584 (5)	
H2	−0.1086	0.4960	0.2916	0.070*	
C3	−0.2296 (5)	0.4392 (3)	0.4410 (2)	0.0816 (8)	
H3	−0.3226	0.5097	0.4391	0.098*	
C4	−0.2224 (5)	0.3437 (3)	0.5320 (2)	0.0848 (8)	
H4	−0.3130	0.3488	0.5907	0.102*	
C5	−0.0814 (4)	0.2415 (3)	0.53555 (16)	0.0681 (6)	
H5	−0.0755	0.1775	0.5972	0.082*	
C6	0.0537 (3)	0.23176 (18)	0.44807 (14)	0.0469 (4)	
C7	0.4681 (3)	0.1852 (2)	0.36729 (17)	0.0589 (5)	
H7A	0.5360	0.2444	0.3986	0.071*	
H7B	0.5927	0.1272	0.3581	0.071*	
C8	0.3767 (3)	0.25404 (16)	0.26244 (15)	0.0435 (4)	
C9	0.0720 (3)	0.38039 (16)	0.16053 (13)	0.0386 (4)	
H9A	−0.0999	0.3767	0.1659	0.046*	
H9B	0.1268	0.3362	0.1106	0.046*	
C10	0.1439 (3)	0.51384 (15)	0.12016 (12)	0.0351 (3)	
C11	0.3619 (3)	0.56739 (15)	0.10351 (14)	0.0399 (4)	
H11	0.5090	0.5274	0.1139	0.048*	
C12	0.4837 (3)	0.79233 (16)	0.04712 (13)	0.0422 (4)	
H12A	0.4078	0.8677	0.0059	0.051*	
H12B	0.6199	0.7737	0.0066	0.051*	
C13	0.5656 (4)	0.81404 (19)	0.14565 (15)	0.0498 (4)	
C14	0.8094 (7)	0.9430 (4)	0.2083 (2)	0.1208 (14)	
H14A	0.6953	0.9796	0.2482	0.145*	
H14B	0.8669	0.8653	0.2541	0.145*	
C15	0.9975 (6)	1.0233 (3)	0.1746 (3)	0.1089 (12)	
H15A	1.0693	1.0380	0.2337	0.163*	
H15B	0.9412	1.1010	0.1303	0.163*	
H15C	1.1128	0.9867	0.1364	0.163*	
N1	0.1656 (2)	0.31606 (12)	0.26288 (10)	0.0359 (3)	
N2	−0.0215 (3)	0.60571 (14)	0.09455 (13)	0.0471 (4)	
N3	0.0854 (3)	0.71393 (14)	0.06368 (13)	0.0486 (4)	
N4	0.3184 (2)	0.69022 (13)	0.06899 (11)	0.0380 (3)	
O1	0.4877 (3)	0.25525 (15)	0.18177 (12)	0.0619 (4)	
O2	0.5302 (4)	0.7457 (2)	0.23075 (13)	0.1013 (8)	
O3	0.6924 (3)	0.91691 (13)	0.12300 (11)	0.0560 (4)	
S1	0.23551 (10)	0.10089 (5)	0.45316 (4)	0.06117 (19)	

### Atomic displacement parameters (Å^2^)

**Table:**

	*U*^11^	*U*^22^	*U*^33^	*U*^12^	*U*^13^	*U*^23^
C1	0.0407 (9)	0.0437 (9)	0.0341 (8)	−0.0073 (7)	0.0014 (7)	−0.0060 (7)
C2	0.0686 (13)	0.0517 (11)	0.0476 (11)	0.0061 (10)	0.0149 (10)	−0.0052 (9)
C3	0.097 (2)	0.0739 (16)	0.0688 (16)	0.0123 (14)	0.0299 (14)	−0.0176 (13)
C4	0.098 (2)	0.104 (2)	0.0478 (13)	0.0025 (17)	0.0262 (13)	−0.0187 (13)
C5	0.0732 (15)	0.0872 (17)	0.0330 (10)	−0.0082 (13)	0.0021 (10)	0.0015 (10)
C6	0.0438 (9)	0.0539 (10)	0.0369 (9)	−0.0068 (8)	−0.0049 (7)	−0.0015 (8)
C7	0.0386 (10)	0.0640 (13)	0.0608 (13)	0.0032 (9)	−0.0069 (9)	0.0052 (10)
C8	0.0369 (9)	0.0409 (9)	0.0481 (10)	−0.0043 (7)	−0.0002 (7)	−0.0046 (7)
C9	0.0370 (8)	0.0419 (9)	0.0338 (8)	−0.0049 (7)	−0.0042 (6)	−0.0049 (7)
C10	0.0326 (8)	0.0403 (8)	0.0291 (8)	0.0007 (6)	−0.0027 (6)	−0.0039 (6)
C11	0.0327 (8)	0.0367 (8)	0.0453 (9)	0.0027 (6)	−0.0022 (7)	−0.0031 (7)
C12	0.0479 (10)	0.0376 (8)	0.0360 (9)	−0.0069 (7)	−0.0020 (7)	−0.0014 (7)
C13	0.0559 (11)	0.0526 (11)	0.0380 (10)	−0.0085 (9)	−0.0009 (8)	−0.0073 (8)
C14	0.153 (3)	0.155 (3)	0.0655 (18)	−0.077 (3)	−0.0210 (19)	−0.039 (2)
C15	0.117 (3)	0.116 (3)	0.096 (2)	−0.047 (2)	−0.038 (2)	−0.0240 (19)
N1	0.0348 (7)	0.0363 (7)	0.0326 (7)	−0.0022 (5)	−0.0014 (5)	−0.0028 (5)
N2	0.0332 (7)	0.0468 (8)	0.0536 (9)	0.0020 (6)	−0.0060 (6)	−0.0009 (7)
N3	0.0394 (8)	0.0432 (8)	0.0555 (10)	0.0059 (6)	−0.0070 (7)	−0.0008 (7)
N4	0.0358 (7)	0.0362 (7)	0.0368 (7)	−0.0004 (5)	−0.0022 (5)	−0.0018 (6)
O1	0.0508 (8)	0.0707 (10)	0.0586 (9)	0.0096 (7)	0.0109 (7)	−0.0119 (7)
O2	0.1505 (19)	0.1102 (15)	0.0355 (9)	−0.0617 (14)	−0.0002 (10)	−0.0028 (9)
O3	0.0673 (9)	0.0560 (8)	0.0456 (8)	−0.0148 (7)	−0.0102 (6)	−0.0131 (6)
S1	0.0544 (3)	0.0533 (3)	0.0578 (3)	−0.0006 (2)	−0.0062 (2)	0.0150 (2)

### Geometric parameters (Å, º)

**Table:**

C1—C2	1.388 (3)	C9—H9B	0.9700
C1—C6	1.399 (2)	C10—N2	1.351 (2)
C1—N1	1.421 (2)	C10—C11	1.362 (2)
C2—C3	1.379 (3)	C11—N4	1.339 (2)
C2—H2	0.9300	C11—H11	0.9300
C3—C4	1.378 (4)	C12—N4	1.448 (2)
C3—H3	0.9300	C12—C13	1.500 (3)
C4—C5	1.365 (4)	C12—H12A	0.9700
C4—H4	0.9300	C12—H12B	0.9700
C5—C6	1.394 (3)	C13—O2	1.191 (2)
C5—H5	0.9300	C13—O3	1.322 (2)
C6—S1	1.751 (2)	C14—C15	1.381 (4)
C7—C8	1.507 (3)	C14—O3	1.446 (3)
C7—S1	1.800 (2)	C14—H14A	0.9700
C7—H7A	0.9700	C14—H14B	0.9700
C7—H7B	0.9700	C15—H15A	0.9600
C8—O1	1.217 (2)	C15—H15B	0.9600
C8—N1	1.363 (2)	C15—H15C	0.9600
C9—N1	1.473 (2)	N2—N3	1.314 (2)
C9—C10	1.495 (2)	N3—N4	1.335 (2)
C9—H9A	0.9700		
			
C2—C1—C6	119.07 (17)	C11—C10—C9	131.21 (15)
C2—C1—N1	120.41 (15)	N4—C11—C10	104.97 (14)
C6—C1—N1	120.48 (17)	N4—C11—H11	127.5
C3—C2—C1	120.2 (2)	C10—C11—H11	127.5
C3—C2—H2	119.9	N4—C12—C13	111.39 (14)
C1—C2—H2	119.9	N4—C12—H12A	109.4
C4—C3—C2	120.8 (2)	C13—C12—H12A	109.4
C4—C3—H3	119.6	N4—C12—H12B	109.4
C2—C3—H3	119.6	C13—C12—H12B	109.4
C5—C4—C3	119.6 (2)	H12A—C12—H12B	108.0
C5—C4—H4	120.2	O2—C13—O3	125.37 (19)
C3—C4—H4	120.2	O2—C13—C12	124.90 (19)
C4—C5—C6	120.9 (2)	O3—C13—C12	109.65 (15)
C4—C5—H5	119.5	C15—C14—O3	112.4 (3)
C6—C5—H5	119.5	C15—C14—H14A	109.1
C5—C6—C1	119.4 (2)	O3—C14—H14A	109.1
C5—C6—S1	120.78 (16)	C15—C14—H14B	109.1
C1—C6—S1	119.84 (15)	O3—C14—H14B	109.1
C8—C7—S1	111.53 (13)	H14A—C14—H14B	107.8
C8—C7—H7A	109.3	C14—C15—H15A	109.5
S1—C7—H7A	109.3	C14—C15—H15B	109.5
C8—C7—H7B	109.3	H15A—C15—H15B	109.5
S1—C7—H7B	109.3	C14—C15—H15C	109.5
H7A—C7—H7B	108.0	H15A—C15—H15C	109.5
O1—C8—N1	121.92 (17)	H15B—C15—H15C	109.5
O1—C8—C7	121.56 (17)	C8—N1—C1	123.85 (14)
N1—C8—C7	116.51 (16)	C8—N1—C9	116.72 (14)
N1—C9—C10	113.91 (13)	C1—N1—C9	119.33 (14)
N1—C9—H9A	108.8	N3—N2—C10	109.05 (14)
C10—C9—H9A	108.8	N2—N3—N4	106.88 (14)
N1—C9—H9B	108.8	N3—N4—C11	111.00 (14)
C10—C9—H9B	108.8	N3—N4—C12	119.89 (14)
H9A—C9—H9B	107.7	C11—N4—C12	128.89 (14)
N2—C10—C11	108.10 (14)	C13—O3—C14	116.59 (19)
N2—C10—C9	120.68 (14)	C6—S1—C7	95.49 (10)

### Hydrogen-bond geometry (Å, º)

**Table:**

*D*—H···*A*	*D*—H	H···*A*	*D*···*A*	*D*—H···*A*
C4—H4···O2^i^	0.93	2.59	3.445 (3)	154

Symmetry code: (i) −*x*, −*y*+1, −*z*+1.
